# Comparing Molecular Mechanisms in Solar NH_3_ Production and Relations with CO_2_ Reduction

**DOI:** 10.3390/ijms22010139

**Published:** 2020-12-25

**Authors:** Domenico Mallamace, Georgia Papanikolaou, Siglinda Perathoner, Gabriele Centi, Paola Lanzafame

**Affiliations:** Departments ChiBioFarAm and MIFT, University of Messina, ERIC aisbl, INSTM/CASPE, V. le F. Stagno D’Alcontres 31, 98166 Messina, Italy; d.mallamace@unime.it (D.M.); gpapanikolaou@unime.it (G.P.); perathoner@unime.it (S.P.)

**Keywords:** molecular mechanisms, N_2_ fixation, NRR, CO_2_-to-C2+, CO_2_RR, electrocatalysis, bioinspired

## Abstract

Molecular mechanisms for N_2_ fixation (solar NH_3_) and CO_2_ conversion to C2+ products in enzymatic conversion (*nitrogenase*), electrocatalysis, metal complexes and plasma catalysis are analyzed and compared. It is evidenced that differently from what is present in thermal and plasma catalysis, the electrocatalytic path requires not only the direct coordination and hydrogenation of undissociated N_2_ molecules, but it is necessary to realize features present in the *nitrogenase* mechanism. There is the need for (i) a multi-electron and -proton simultaneous transfer, not as sequential steps, (ii) forming bridging metal hydride species, (iii) generating intermediates stabilized by bridging multiple metal atoms and (iv) the capability of the same sites to be effective both in N_2_ fixation and in CO_x_ reduction to C2+ products. Only iron oxide/hydroxide stabilized at defective sites of nanocarbons was found to have these features. This comparison of the molecular mechanisms in solar NH_3_ production and CO_2_ reduction is proposed to be a source of inspiration to develop the next generation electrocatalysts to address the challenging transition to future sustainable energy and chemistry beyond fossil fuels.

## 1. Introduction

N_2_ fixation to directly produce ammonia by using solar energy (solar NH_3_) is an emerging reaction of great interest. It is the sustainable alternative to the current industrial large-scale Haber–Bosch (HB) process of ammonia synthesis requiring the use of a fossil fuel as a H_2_ source and high temperatures/pressure to convert N_2_ to ammonia [1,2,3,4,5,6,7]. The HB process has been largely improved over the last century, reducing the minimum energy input from about 60 GJ·t_NH3_^−1^ to the current values ranging between 27 and 32 GJ·t_NH3_^−1^ [7]. However, ammonia production remains a main chemical process responsible for greenhouse gas (GHG) emissions, accounting for about 1.2–1.5% of the total global GHG emissions and over 350 Mtons of CO_2_ emissions yearly.

There are three technology generations which can reduce this impact. The first generation uses CO_2_ sequestration (blue rather than gray hydrogen). The carbon sequestration adds energy costs and thus the effective CO_2_ equivalent emission reduction is lower than 30%. As it is a technology that is already available (but with added costs and complexity), it could be used in the existing production, not expanding the current uses of ammonia. Ammonia is an interesting energy and hydrogen vector, with advantages over alternative vectors in terms of the amount transported by weight and the avoidance of transporting the carrier back (the N_2_ produced is directly released to the atmosphere). The advantages of NH_3_ are the low cost per unit of stored energy (half a year of ammonia storage would cost 0.54 USD/kg H_2_ compared to 14.95 USD/kg H_2_ of pure H_2_ storage [8]), higher volumetric energy density (7.1–2.9 MJ/L), easier and more widespread production, consolidated handling and distribution capacity and better commercial viability. Ammonia was defined as a game changer [8]. However, the extension of ammonia out of the chemical (mainly fertilizers) sector to become a key enabling element for a future energy (low-carbon) scenario would require the development of improved ways to produce ammonia directly from N_2_.

The second-generation HB process [1] is a multistep process, with (i) production of renewable energy (RE), (ii) water electrolysis to produce H_2_ (green hydrogen) and then (iii) use of the hydrogen in a conventional catalytic hydrogenation of N_2_ (high temperature/pressure). The technology has already been demonstrated on a pilot scale, by Siemens, for example, and spin-off companies are already proposing it on the market, for example, the SME Proton Venture in Netherlands. Inefficiencies derive from the (i) need for multiple steps, (ii) the coupling of a high temperature/pressure catalytic step to an electrolyzer step operating under very different conditions and dynamics and (iii) losing energy derived from overpotentials in producing H_2_ molecules and in their activation to react with nitrogen and generate ammonia.

H^+^/e^−^ pairs are the equivalent of H_2_, but are the primary species generated from water oxidation in photoelectrocatalytic processes. Their direct use avoids the energy losses related to the production/use of H_2_. In addition, in a photoelectrocatalytic (PEC) devices, all the stages from solar light adsorption to redox reactions, leading to ammonia generation from N_2_ and H_2_O, are integrated in a single device. This makes possible the development of compact artificial leaf-type devices able to produce in a distributed way ammonia as a vector to transport renewable energy or H_2_, or to produce fertilizers in a decentralized way. This could be considered the third-generation HB process. However, an intermediate stage between the second and third generation is the development of electrocatalytic devices (driven from renewable electrical energy). This is the area where most of the recent research on N_2_ fixation (often indicated as N_2_ reduction reaction, NRR) is focused, with the direct photocatalytic reduction of N_2_. A selection of recent reviews on these aspects are presented in [1,2,3,4,5,6,7,8,9,10,11,12,13,14,15,16,17,18,19,20].

However, there are other ways for N_2_ fixation to produce ammonia, also based on RE. Renewable electrical energy could generate non-thermal plasma (NTP), which is a (partially) ionized gas, consisting of neutral species (molecules, radicals, excited species), ions, photons and electrons [21]. In NTP, the electron temperature is largely higher than the temperature of the other species and thus the radicals and excited species are formed close to ambient temperature. Combined with a catalyst (plasma catalysis [22,23]), NTP is a valuable alternative to conventional catalysis, because it directly generates activated species under ambient conditions. The control of the selective path of transformation derived from the presence of a catalyst allows us to explore new paths to produce chemicals by using renewable energy sources. Plasma catalytic ammonia synthesis thus represents an alternative to photo- and/or electro-catalytic routes [24,25].

On the other hand, the enzymatic conversion of N_2_ to NH_3_ with *nitrogenase* is the reference for a sustainable N_2_ fixation indirectly using solar energy. However, from a practical perspective, energy consumption in the form of adenosine triphosphate (ATP) is required and to synthesize the reducing agents and proton sources, the overall energy for nitrogen fixation is higher than that for the HB process [26]. However, in terms of reaction mechanism [27], it remains a valuable reference to better understand the possibilities for N_2_ fixation in ambient conditions, as occurs in photo- and/or electro-catalytic processes and in plasma catalysis.

A comparison between the different molecular mechanisms of N_2_ fixation could offer a better understanding of analogies and differences, and thus inspire better designed systems. Notwithstanding the many reviews on N_2_ fixation, a better comparison between the mechanisms of these different approaches would be useful.

There is, in addition, a further interesting aspect and motivation for this comparison. *Nitrogenase* can reduce CO_x_ to hydrocarbon-forming compounds such as ethylene, ethane, propylene and propane [28,29,30]. This is also unique for this enzymatic system. The photo-/electro-catalytic reduction of CO_2_ (CO_2_RR) to form >C1 products is an attractive reaction for the possibility to build a new value chain in the conversion of CO_2_ and form high-added-value chemicals and fuels [31]. Directly producing C2-C3 olefins or oxygenated compounds from CO_2_ would provide a direct path to overcome the use of fossil fuels [32]. There is a large debate in the literature on the molecular mechanisms of C-C formation in CO_2_ photo-/electro-catalytic conversion and discordant hypotheses [33,34,35,36,37,38]. In NRRs, there are also dissimilar proposed reaction mechanisms and natures of the active sites, despite the relatively limited variations in the performances [39].

An analysis and comparison of the molecular mechanisms of solar NH_3_ production and CO_2_ reduction to >C1 products, particularly in systems able to give both reactions, by using *nitrogenase* as a reference of a molecular system able to perform both reactions, could thus provide clues for a better interpretation of the mechanistic aspects of these challenging reactions.

## 2. Mechanisms in *Nitrogenase*

**Figure 1:**
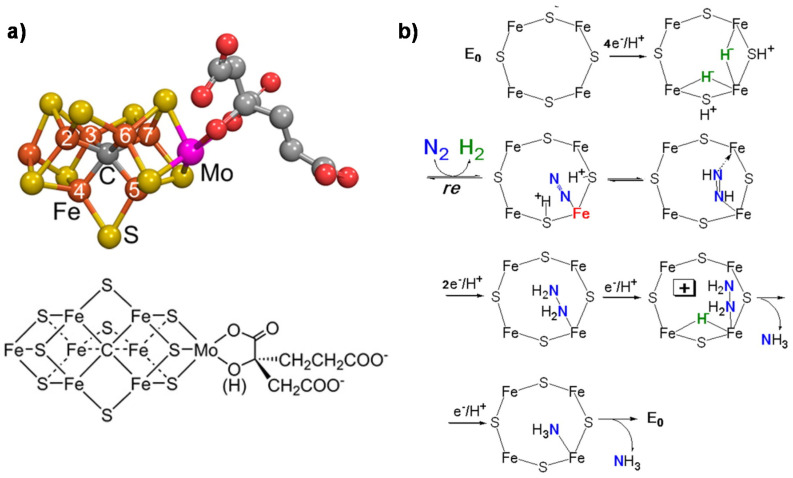
(**a**) FeMo cofactor (Fe in orange, Mo in magenta, S in yellow, C in gray and O in red); (**b**) mechanism (key elements) of N_2_ fixation on FeMo-co. Adapted from [27]. Not subject to US copyright.

*Nitrogenase* is a unique system able to convert N_2_ to NH_3_ [27]. Total cost of N_2_ reduction corresponds to eight electrons being transferred and 16 MgATPs being hydrolyzed. Three classes of *nitrogenase* differ in the heteroatom present in the active site metal cluster (Mo, V or Fe), but the Mo-dependent nitrogenase is the most important and best-studied enzyme [40]. It contains two metallo-components, *dinitrogenase* (molybdenum–iron (MoFe) protein) and *dinitrogenase reductase* (Fe protein), which associate and dissociate in a catalytic cycle also requiring a reducing source and MgATP [41]. The MoFe protein contains two metal clusters: the iron–molybdenum cofactor (FeMo-co) [42], which provides the active site for substrate binding and reduction, and a P-cluster, involved in electron transfer from the Fe protein to FeMo-co [43]. The FeMo cofactor is thus the key element for the mechanism of N_2_ fixation. It also contains an interstitial carbon atom [44] which is key for the CO_2_ conversion mechanism, providing stability to the complex. Figure 1a reports the model of the FeMo cofactor, while Figure 1b shows the proposed mechanism of N_2_ fixation [27].

The key features of this mechanism [45,46] are:

(i) A specific binding site for N_2_ able to first accept four electrons/protons to form two [Fe–H–Fe] bridging hydrides,
(ii) coordination of N_2_ on two iron atoms with simultaneous reductive elimination of H_2_,
(iii) multi-electron/proton transfer to a coordinated undissociated N_2_ molecule to form a N_2_H_2_ molecule stabilized by interaction with two iron atoms,
(iv) further multi H^+^/e^−^ transfer to form an end-on N_2_H_4_ coordinated molecule,
(v) further steps of H^+^/e^−^ transfer with stepwise release of ammonia.

The simultaneous electron/proton transfer to an undissociated coordinated N_2_ molecule is a different feature regarding the mechanisms in iron catalysts for the high-temperature/pressure HB process, where the first (rate-limiting) step is the dinitrogen dissociative chemisorption followed by sequential hydrogenation of the intermediates [47,48,49,50]. The reason for the difference in the mechanism is that the nitrogen molecule dissociation path, in principle, is preferable as sequential steps of electron transfer and hydrogenation of the nitrogen adatoms. However, the strongly bound nitrogen species formed by dissociation require high temperatures to avoid catalyst inhibition, and, therefore, there is a need for high-pressure operations (as this is an exothermic reversible reaction). For the undissociated N_2_ molecule activation, sequential electron and proton transfer steps would generate high-energy intermediates [51]. For example, adding one electron and one proton to N_2_ to form a N_2_H* species requires −3.2 V vs. normal hydrogen electrode (NHE). For multi-electron reduction processes, for example, to produce N_2_H_5_^+^ adspecies, the reduction potential becomes −0.23 V. Multi-electron reactions are thus necessary in the low-temperature profile, in agreement with what was observed for *nitrogenase*, and to avoid/minimize the side reaction of H^+^/e^−^ recombination to form H_2_, which is also possible in the enzyme path.

**Figure 2:**
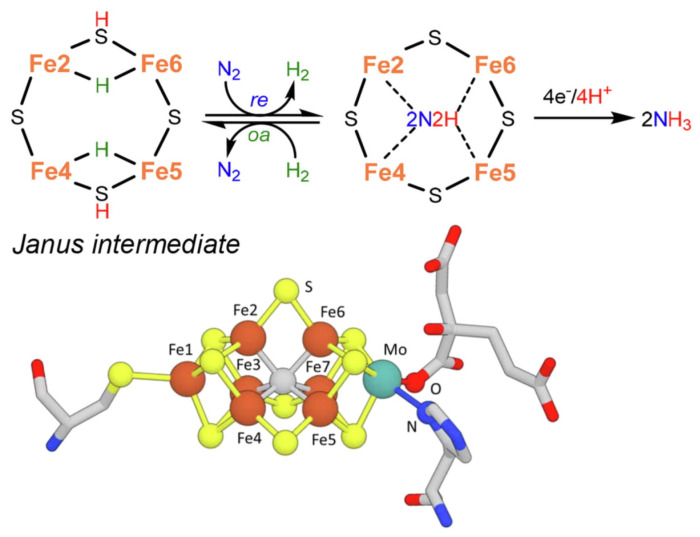
Key step for biological N_2_ fixation and structure of nitrogenase FeMo-co. Adapted from [52]. Copyright 2021 Elsevier.

As a slight change, more recent indications in the mechanism [52] instead suggest the formation of a Janus intermediate, i.e., the formation of a symmetric hydride structure with a subsequent diazene intermediate (Figure 2).

**Figure 3:**
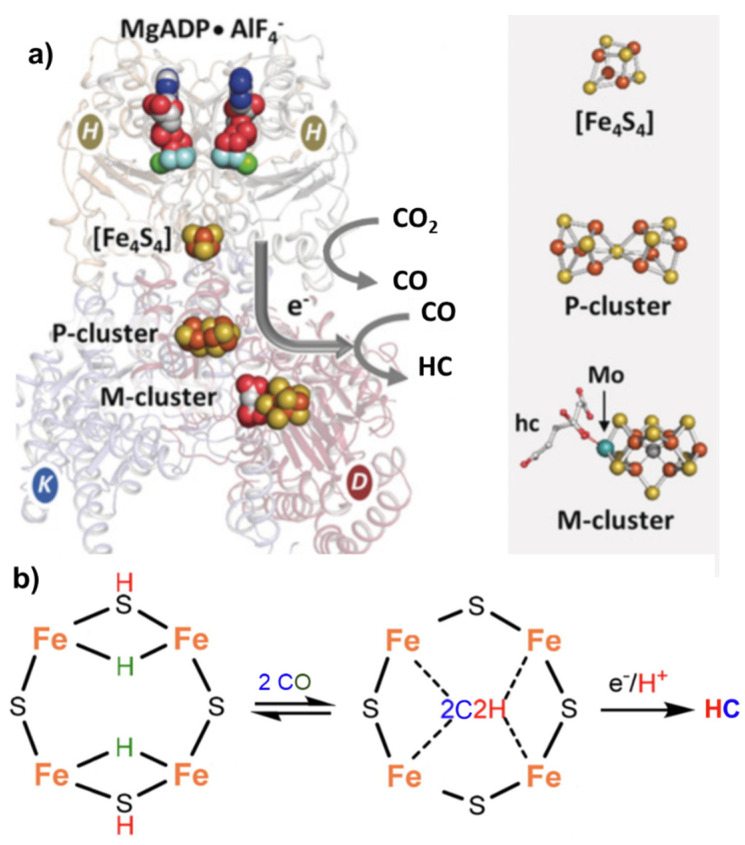
(**a**) Nitrogenase enzyme with indications of the key components involved in electron transfer, including MgADP AlF_4_−, [Fe_4_S_4_] cluster, P-cluster and M-cluster with indication of the active centers involved in CO_2_ to CO and CO_2_ to hydrocarbon (HC) conversion. Adapted from [28]. Copyright 2016 Wiley. (**b**) Proposed schematic mechanism of the active sites in FeMo cofactor (M-cluster) for conversion of CO to HC.

*Nitrogenase*, particularly in the vanadium form [53], can also catalyze the reductive carbon–carbon coupling of COx into hydrocarbon products, as indicated before. This is also a unique feature and suggests a role of this enzyme as an evolutionary link between the nitrogen and carbon cycles on Earth [28]. The mechanism of C-C coupling is not clear, but progress has been made recently [54]. From CO_2_, the mechanism involves a first step of reduction of CO_2_ to CO by the Fe protein ([Fe_4_S_4_]^0^) of *nitrogenase* [55], followed by the reductive carbon–carbon coupling of two coordinated CO molecules on the same active site of the *nitrogenase* cofactor proposed for N_2_ fixation (M-cluster). The tentative mechanism is presented in Figure 3.

**Figure 4:**
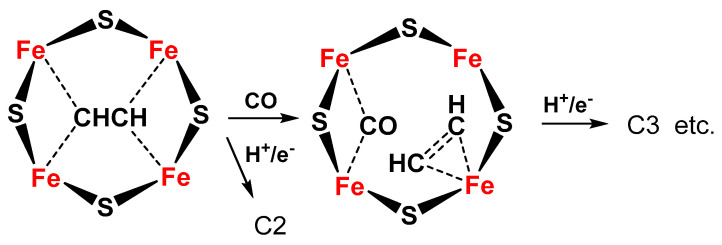
Proposed schematic mechanism of the active sites in FeMo cofactor for conversion of C2 intermediate to C3 products (hydrocarbon, oxygenates) via formation of a ferracycle species.

The same Janus intermediate proposed for N_2_ fixation (symmetric hydride structure) reacts with two CO molecules (produced on the Fe protein) to form an ethyne-like intermediate which can be hydrogenated to ethylene or could react further to form a ferracycle (formed by C_2_H_4_ binding to one of the Fe atoms [56]), leaving the other Fe free to form other Fe-hydride species and further coordinate CO, which can react with the ferracycle species, to give C2 products (hydrocarbon, oxygenated chemicals) (Figure 4).

## 3. Bioinspired Approaches

Several bioinspired approaches have been developed to mimic the nature of the active centers present in *nitrogenase* [52,57]. As commented above, the active site (E_o_ in Figure 1b) able to host the dinitrogen binding after the initial formation of the dihydride species (the Janus intermediate in Figure 2) possesses vacant coordination sites [54]. Bioinspired mechanistic studies have thus focused on producing coordinatively unsaturated iron centers for N_2_ binding and the formation of hydrides. However, the key mechanistic issue evidenced before, e.g., the presence of a simultaneous multi-electron/proton transfer, has rarely been considered. Four electrons and four protons must be accumulated on the FeMo-co resting state to generate the Janus intermediate (Figure 2) responsible for N_2_ binding and conversion. The Janus intermediate contains two [Fe-H-Fe] units and two protons on bridging sulfides [58]. Four coordinatively unsaturated iron sites cooperate in accumulating electrons/protons as bridging hydrides, whilst avoiding changes in the formal oxidation state of the metal core. Dihydrogen release in a reductive elimination pathway provides the two remaining reducing equivalents for N_2_ coordination and the first stage of its reduction to form a diazenidometal complex.

Note, however, there are still open questions regarding the N_2_ fixation mechanism, such as (i) the sequence of proton/electron transfers on the N_2_ unit, (ii) when ammonia release occurs (distal and alternating mechanistic pathways [59]) and (iii) if and how the molybdenum center [60] and the central carbide [61] influence the reactivity of the FeMo-co for N_2_ binding and reduction. This is a likely role of the central carbide in stabilizing the complex and coordinating the diazene or ethyne intermediate. However, these could be considered secondary aspects in terms of developing molecular complexes analogous to the *nitrogenase* active centers.

The bioinspired metal complexes reported in the literature focused on the coordination of N_2_ molecule and sites (especially metal hydride) able to hydrogenate the coordinated nitrogen molecule. Several molecular coordination complexes show activity in N_2_ binding and reduction [62,63,64]. Starting from the first example of systems for N_2_ fixation and reduction, based on single molybdenum [65] or iron [66] centers, many other metal complexes have also been developed [62], including the use of different metal centers such as V, Co, Ru, Os, W and Ti. Note that mechanistic studies refer typically to discrete e^−^ and H^+^ transfer steps, although concerted proton-coupled electron transfer (PCET) steps have not been excluded (even if their occurrence is rarely demonstrated). These mononuclear metal complexes were not active for multi-electron/proton transfer. The studies were focused on the synthesis and mechanistic aspects, rather than on the performances. Several of these metal complexes show some activity in the conversion of N_2_ to NH_3_, although typically under conditions of difficult practical utilization. The rates of reactions and efficiencies were low. Few, and mainly supported phthalocyanines, were tested under electrocatalytic conditions, but not showing relevant performances [62]. Thus, while these studies provided good mechanistic insights, the advances in terms of the development of effective catalytic systems for N_2_ to NH_3_ conversion were limited. These studies on mononuclear metal complexes did not reproduce the main key features of *nitrogenase,* as multi-metal centers and, therefore, a multi-electron/proton mechanism, were not present, although the sequence of the hydrogenation of undissociated N_2_ is typically present. End-on coordination of N_2_ is the most commonly considered situation, but two mononuclear complexes can operate in synergy (pairing) to coordinate the N_2_ molecule in a bridging mode.

The activation of dinitrogen by polynuclear metal complexes was reviewed recently by Singht et al. [63]. To circumvent the low reactivity of N_2_ (high reductive potential for one electron reduction, low proton affinity and high ionization potential of dinitrogen), and weak interaction with transition metal ions (lack of a dipole moment and relatively high-energy π* orbitals result in N_2_ being a poor σ-donor and π-acceptor), the mononuclear systems strategy is based on reducing metal centers (i.e., formal Fe^0^ centers), while in multinuclear systems, it is possible to coordinate N_2_ in a bridge mode to weaken the *n* ≡ *n* bond and thus make nitrogen susceptible to protonation.

**Figure 5:**
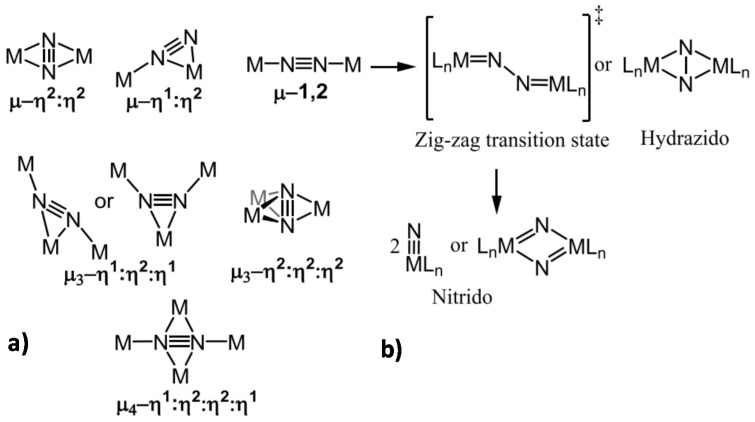
(**a**) Binding bridging modes of N_2_ in polynuclear metal complexes. (**b**) Proposed dimetallic N_2_ scission mechanism. Adapted from [63]. Copyright 2020 American Chemical Society.

Different modalities of dinitrogen-derived bridging ligand(s) coordinated to multiple redox-active centers have been validated experimentally [63] and Figure 5a,b instead reports the possibilities to weaken the *n* ≡ *n* bond-forming diazenido-, hydrazido- or nitrido-metal species. This is thus an alternative to activate N_2_ molecules in terms of a proton-coupled electron transfer (PCET) mechanism.

There are different examples of N_2_ activation by multi-metallic complexes [63]. Mostly, however, they are stoichiometric complexes and not involved in a catalytic cycle. How to translate this wide body of knowledge on the cooperative N_2_ activation in multi-metallic systems to develop effective catalysts for N_2_ fixation (besides the often-existing problem of cost and stability of the proposed complexes) is still a challenge. In comparison with the *nitrogenase* mechanism, it may be commented that the formation of multi-metal-bound intermediates is part of the mechanism, which typically occurs simultaneously with hydrogen transfer to the coordinated N_2_, differently from what studies on these multi-metallic complexes show.

Transition metal–sulfur (M-S) compounds are a third class of inorganic systems investigated for N_2_ activation, and they clearly can be considered as directly bioinspired, starting from the active centers present in *nitrogenase*. Three classes of M−S compounds were reviewed by Tanifuji and Ohki [64]: (i) multinuclear M−S clusters structurally or functionally relevant to the *nitrogenase* cofactors, (ii) mono- and dinuclear transition metal complexes supported by sulfur-containing ligands in N_2_ and N_2_H_x_ (x = 2, 4) chemistry (thus mimicking intermediates in the mechanism of N_2_ fixation) and (iii) metal sulfide-based solid materials employed in the reduction of N_2_. However, only for the latter class have reactivity data been reported, while there is less information on the molecular mechanisms and thus insights on how to translate the mechanistic indications to solid materials provided by the *nitrogenase* enzyme. Thus, Tanifuji and Ohki’s [64] indications were that “with regard to the reactivity studies of M−S clusters, catalytic N_2_ conversion still remains a challenge, while substoichiometric N_2_ reduction has been recently achieved.”

This short analysis of the studies on N_2_ activation and bioinspired approaches in metal complexes indicates that notwithstanding the large synthetic and characterization effort, the indications on how to design robust and active solids which can reproduce enzymatic mechanisms of N_2_ to NH_3_ conversion remain elusive.

## 4. From Electrocatalysis to Biomimicking Mechanisms

The previous section showed how the state-of-the-art approaches in biomimicking metal complexes, although active in N_2_ coordination and, in part, in the further conversion to NH_3_, do not describe the key features of the proposed mechanism for *nitrogenase* well, neither its ability for both N_2_ fixation nor its ability to give products with C-C bond formation (indicated as C2+ products) in CO_x_ conversion. In addition, the realization of a full catalytic cycle for N_2_ to NH_3_ conversion with high turnover number (TON) is still a challenge. It is thus worth looking whether, among the existing electrocatalysts, there are systems active in both N_2_ fixation (NRR) and the formation of >C1 products in CO_2_ conversion (CO_2_RR), and which have features which may resemble those of the active sites of *nitrogenase*.

A relevant example is given by electrocatalysts based on iron oxide on carbon nanotubes (CNTs, multiwalled). These electrocatalysts were among the first identified to be active in the formation of >C1 products (hydrocarbon, oxygenated chemicals) in CO_2_ electrocatalytic conversion, particularly in isopropanol synthesis, with a challenging reaction being an 18e^−^ reduction [67,68]. Note that earlier studies and several theoretical studies instead indicated that the formation of C2 products in the electrochemical conversion of CO_2_ is only possible over particular copper faces (the {100} face) [69,70]. In these copper systems, the involvement of coupling reactions between surface adspecies is suggested as the path to produce multi-carbon products, by the dimerization of two chemisorbed CO molecules on metallic copper [71,72], a chemisorbed CO and other intermediates, such as CHO [73], or the reaction of chemisorbed CO with adsorbed acetaldehyde (H_3_C-C=O) to form C3 products (1-propanol) [37]. Chan et al. [74] suggested that Cu(100) planes with surface strains induced by compression and elongation of perpendicular axes are geometrically beneficial for C2 product formation; by stabilizing the CO in bridge sites, they infer a low activation energy for CO–CO coupling. Note that all these mechanistic indications refer to metal surfaces, and not to oxide nanoparticles, while there are several indications that the oxide or derived one (such as hydroxide) are the active species. In addition, no systems reported as active in CO_2_ electrochemical conversion to >C1 hydrocarbons or oxygenated compounds are also active in N_2_ fixation, differently from the iron oxide on CNT electrocatalysts [75,76].

Recent results [77] have demonstrated that the active phase in these systems for N_2_ fixation is a metal oxide, at least in the ex situ situation, because operando near edge X-ray absorption fine structure (NEXAFS) results combined with ambient pressure XPS data show the in situ formation of FeOOH species stabilized at carbon defect sites during the electrocatalytic reaction [78].

During N_2_ fixation, the Fe_2_O_3_/CNT electrocatalyst transforms in situ. After 3 h of operation, the electrocatalyst is removed and washed, most of the iron oxide (weakly interacting with the carbon support) could leave only small (2 nm) iron oxide nanoparticles, sitting at defects of CNTs [79]. Their characterization showed they have been transformed in situ to a maghemite (-Fe_2_O_3_) structure regarding the initial hematite (-Fe_2_O_3_) structure. This transformation leads to an about five times increase of both ammonia formation rate and Faradaic selectivity to ammonia, likely due to the in situ formation of a γ-FeOOH rather than α-FeOOH nanostructure (as for hematite).

**Figure 6:**
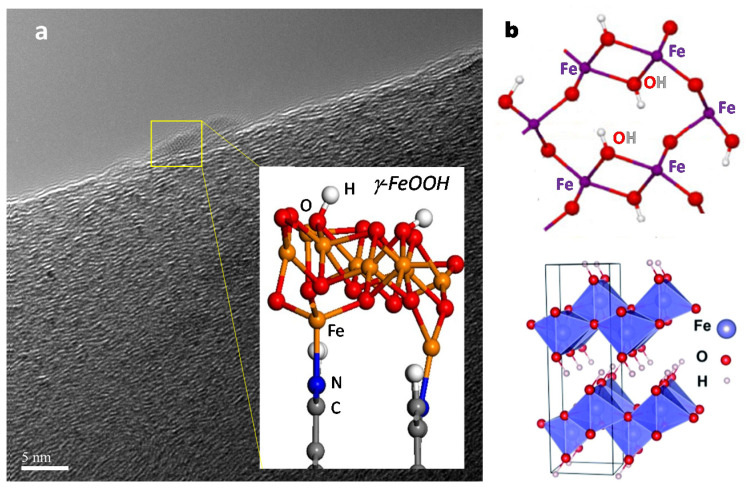
(**a**) High-resolution transmission electron microscopy (HRTEM) of activated iron-oxide/carbon nanotube (CNT) electrocatalysts for N_2_ reduction reaction (NRR) (adapted from [79]), with the inset reporting a lateral view of the optimized ferrihydrite nanostructures decorating the N-doped graphitic zigzag edges (adapted from [78]. Copyright 2018 Springer Nature). (**b**) Proposed γ-FeOOH (bottom) nanostructure and related surface structure (top) for simultaneous multi-electron and -proton transfer in NRR and CO_2_RR (for the latter, to >C1 products). Adapted from [31]. Copyright 2019 Elsevier.

Figure 6a reports the proposed optimized FeOOH/N-C interface nanostructure (where N-C indicates the N-doped nanocarbon) which forms by the application of a negative potential within the range of relevance for the electrocatalytic behavior. The relevance of the specific nanostructure derives from the observation, as remarked in Figure 6b, of strong analogies between the surface arrangement in γ-FeOOH [31] and the suggested mechanism of reductive conversion of N_2_ in the *nitrogenase* FeMo cofactor discussed above. Although S atoms are not present, the surface structure of γ-FeOOH presents biomimetic sites able to give a concerted multi-electron and -proton reductive transfer mechanism and N_2_ or CO coordination, as for those reported in Figure 2 (N_2_ fixation) and Figure 3 and Figure 4 (CO_2_ reduction to multi-carbon species).

Note that these sites, different from other hypotheses on molecular mechanisms for both NRR and CO_2_RR (to >C1 products), could well explain why these electrocatalysts are active in both reactions as *nitrogenase*. The application of the mechanism proposed for C3 products in *nitrogenase* (Figure 4) could also explain why experimentally the formation of isopropanol is observed well, while theoretical approaches predict the formation of the linear C3 alcohol (1-propanol) [37]. The model of the active sites outlined in Figure 6b agrees with the experimental observation that the oxide is the active species in the electrocatalytic reaction rather than the metal or a nitride species [77].

Although the model proposed in Figure 6b is not proven, except in terms of in situ formation of a FeOOH species during the electrocatalytic reaction [78], the point remarked on above supports this hypothesis and stimulates the need to explore this possibility of biomimetic electrocatalysis in more depth. There is the need to prove and better identify the nature of these surface sites, and especially investigate how to enhance their formation and to increase the performances, which, however, are already among the best reported values for both N_2_ fixation and CO_2_RR to >C1 products. There is an undoubted role of carbon defects in the stabilization of this species, and the high-resolution transmission electron microscopy (HRTEM) image presented in Figure 6a shows that the iron-oxide/hydroxide nanoparticles are sitting at carbon defects.

Thus, although different reaction mechanisms could also be possible for both NRR and CO_2_RR, the mechanism suggested based on biomimetic sites as those present in *nitrogenase* and able to (i) give multi-electron and -proton transfer, (ii) chemisorb N_2_ and vicinal CO molecules (bridging multiple Fe atoms) and (iii) generate close hydride species also bridging Fe atoms, which is attractive for the possibility to explain many experimental features, at least on these electrocatalysts.

## 5. Other Proposed Molecular Mechanisms of Reaction

A large variety of reaction mechanisms and the associated nature of the active sites have been proposed in the literature. To focus discussion, we limit ourselves here to the analysis of suggested hypotheses, typically based on a DFT (Density-functional theory) theoretical approach, for nanocarbon-based electrocatalysts for NRR [39]. This analysis represents the general state-of-the-art of the sector. Note that results reported in the literature for N_2_ fixation at ambient temperature and pressure, using H_2_O as the hydrogen source, typically range between 0 and 20% Faradaic selectivity and between 0.05 and 0.2 of current density to ammonia (J_NH3_, mA/cm^2^). Although there are variations, industrial targets should have a Faradaic selectivity higher than 80% and specific current densities higher than 20–50 mA/cm^2^ [39]. From this perspective in terms of industrial targets, all reported results are similar and hardly justify a very large range of proposed reaction mechanisms with different natures of the active sites.

A non-exhaustive list of the proposed natures of the active centers in N_2_ fixation:

1. presence of C=O and O-C=O groups in graphene sheets [80];
2. electron-deficient environment at the B-doping positions in boron-doped graphene [81];
3. P atoms substituting C atoms in phosphorus-doped carbon nanotubes [82];
4. pyridinic and pyrrolic N in N-doped porous carbons [83];
5. defective nature of carbon-doped boron nitride nanosheets [84];
6. sp^2^-hybridized B, due to substitution of an edge N atom in the cavity of C_2_N [85];
7. double boron atom species in defect cavities of C_2_N [86];
8. atomically dispersed Ni sites in a carbon matrix [87];
9. heteronuclear dual-atom catalytic elements in FeMo/g-C3N4 [88];
10. iron atoms in iron-phthalocyanine (FePc) grafted on oxygen-functionalized multiwalled carbon nanotubes (O-MWCNTs) [89];
11. oxygen vacancies in NiCo_2_O_4_ on hollow N-carbon polyhedra [90];
12. copper atoms on activated carbon functionalized with sulfonated groups [91].

It is evident from the above indications that a very large variety of hypotheses on the active sites in NRR have been proposed (this is limited to only nanocarbon-based electrodes), even though performances were in a relatively narrow range and far from the industrial need, as commented above. The nature of the active sites ranges from sites related to modifications of the nanocarbon itself (defect sites, carbon atoms with charge density due to strains and/or defects, different type of heteroatoms, isolated metal ions or metal dimers inserted in carbon defects) to supported metal atoms or nanoparticles.

From the perspective of biomimetic systems of nitrogenase, none of the above electrocatalysts can be considered to have the key features of the active sites in the enzyme, and even if not specifically investigated, the systems were not among those reported to be able to convert CO_2_ electrocatalytically to >C1 products (multi-carbon) and thus able to perform the reaction of carbon–carbon coupling during electrocatalysis.

The iron oxide/nanocarbon system discussed in the previous section thus remains a unique system in terms of the capability for good performances in both NRR and CO_2_RR (to >C1 products) as in nitrogenase, with sites that can mimic those in nitrogenase, even if their catalytic role was only indirectly proven.

In multi-carbon CO_2_RR, a large range of mechanistic hypotheses have also been indicated sometimes based on theoretical modeling [37,72,73,74,92,93,94,95,96,97,98,99,100,101]. The proposals are different in terms of the mechanism, type of intermediates and active sites. For example, Han et al. [101] indicate Cu catalytic sites sitting at defects of N-doped porous carbon materials as being effective. The pyridinic N species react with CO_2_ to produce CO and, with Cu catalytic sites, act cooperatively to produce C_2_H_5_OH and C_3_H_7_OH via a two-site mechanism. Marepally et al. [100] analyzed the behavior of Cu- and Fe-based dendritic-type electrodes and reported that the nano-morphology positively influences not only the activity, but especially the selectivity to >C1 products (i.e., ethanol, isopropanol, acetic acid) when smoother edges and denser surface sites are present. Genovese et al. [99] indicate a mechanism of >C1 formation (acetate) involving the reaction between CH_x_ adspecies (formed by reduction of CO_x_) on copper nanoparticles and the carbon dioxide anion radical (formed by one electron transfer to CO_2_) present in the double layer. Lum et al. [98] indicated that high selectivity to C2+ products is attained in overpotential conditions (−1 V vs. RHE) where the density is sufficiently high to induce (due to mass diffusion limitations) an increase in the pH near the catalyst surface. It is thus not a role of specific active sites, but of controlling the local conditions (three-phase boundary, pH, etc.) at the electrocatalyst/electrolyte interface. Sen et al. [97] indicated that surface confinement (in copper nanofoams), and, thus, again, a change of the local situation at the electrocatalyst–electrolyte interface, are the key for C2+ products. Zhang et al. [96] also indicated that Cu_2_O nanocavities are necessary for CO_2_ reduction to ethanol through the confinement of the CO intermediate. Zhuang et al. [95] also indicate Cu_2_O nanocavities as necessary to confine the intermediates and allow the formation of C2+ products.

These authors thus emphasize the confinement of intermediates near the surface as the key aspect to form multi-carbon products. Other authors instead indicate the need to have specific surface sites on the electrocatalyst. Li et al. [94] indicated that CO_2_ to C2+ conversion requires an enriched disorder in the copper compared to crystalline Cu, obtained by electrochemical nanocrystal scrambling, i.e., the dynamic fusion of Cu nanoparticles under CO_2_ reducing conditions to form multi-carbon active scrambled nanocrystals. It is not well described which new type of active sites forms in this scrambling mechanism, or the influence on the mechanism, but it is hypothesized that this leads to a higher population of strongly bound *CO (the asterisk indicates a surface-bound species), due to disorder-induced microstrain that alleviates the undesired repulsive interactions of a high density of bound *CO [102], comparable to the creation of optimal geometries in enzymatic systems.

These mechanisms indicate the need to have a metallic Cu surface to form C2+ products, and analogously, Munir et al. [93] indicated that C–C coupling is favored on Cu^0^ sites rather than Cu_2_O. Chan et al. [74] modeled copper(100) faces and found that surface strains with one compressed axis and one elongated axis are geometrically beneficial for C2 product formation. The surface strain stabilizes the CO binding on the bridge sites (*CO_bridge_) and the C2 intermediates, *OCCOH and *OCCO, which maintains a low activation energy of CO–CO coupling. The surface strain also suppresses *H formation. These sites are also active in C3 formation. Goodpaster et al. [73] indicated that Cu(100) is necessary for the mechanism of C–C bond formation through a CO dimer at low potential (formed at adjacent Cu sites), while at high potential C–C bond formation occurs through a reaction of adsorbed CHO and CO, forming an OCCHO_ads_ species (intermediate also in the CO dimer path), which is further hydrogenated to ethylene.

A group of other authors instead indicate that oxide or hydroxide species are those active in CO_2_ electrocatalytic conversion. Eilert et al. [92] indicated that Cu_2_O nanocubes or copper(II)–carbonate/hydroxide are the active species in multi-carbon bond formation. Chang et al. [37] indicate Cu_2_O-derived Cu as the active element, with the C–C coupling mechanism between CO and surface-bound acetaldehyde as the key step to form C3 (1-propanol), while hydrogenation of the acetaldehyde gives the C2 alcohol (ethanol). The surface-bound acetaldehyde is indicated as the intermediate reported in the literature [103], even though this indication is questionable. Results indicate an enol-like surface intermediate (on the metallic Cu surface) involved in the formation of multi-carbon products.

Even if this short survey is not exhaustive of the many indications of the surface mechanisms in the electrocatalytic conversion of CO_2_ to C2+ (multi-carbon) products, there are discordances on the active sites, and even in the surface oxidation state. Most papers claim a unique behavior for copper electrocatalysts, but iron-based catalysts (oxide/hydroxide) behave even better in forming C2+ products [67,68,104,105] and even Pt, a metal typically considered unable to give C2+ in the electrocatalytic conversion of CO_2_, may reach selectivity to C2+ > 60% under specific conditions of reaction [106,107]. The proposed mechanism and nature of the active sites cannot explain a single type of mechanism/active site able to explain the behavior both in N_2_ fixation (NRR) and CO_2_ reduction to multi-carbon products, as present in nitrogenase and the supported iron catalysts described in the previous section.

Although different mechanisms and natures of the active sites could exist, the search for systems active in both reactions which mimic nitrogenase is a valuable direction to develop these electrocatalysts and make a breakthrough advance in the field.

## 6. Molecular Mechanisms in Plasma Catalysis

As an introduction to this section, it should be clarified there are two broad categories of plasma, thermal and non-thermal. Thermal (equilibrium) plasma is a partially ionized gas with the temperature of the charged particles close to that of the neutral particles, thus of the order of 1000–3000 K. Non-thermal (non-equilibrium) plasma (NTP) is instead characterized by the smaller charged species (electrons) having a temperature several orders of magnitude higher than that of neutral molecules. Using the NTP approach, it is possible to generate high-energy species which can activate molecules while keeping the reaction temperature and energy consumption low. In this way, excited dinitrogen species (N_2_*) obtained by collisions with highly energetic electrons can be obtained, for example, via this reaction:

e^−^ + N_2_ → e^−^ + N_2_* (1)

Once formed, excessive energy stored in vibrational modes may be efficiently provided for chemical reactions. This is typically a gas-phase chemistry. H_2_O should be used rather than H_2_ to make the process more sustainable and reduce costs. However, yields of ammonia and energy efficiencies are still unsatisfactory [21,24,108], although they strongly depend on the plasma reactor configuration and mode of operation (pulsed plasma, for example). In terms of process design, it could be preferable to form NOx from N_2_ in a NTP reactor and then reduce NOx to ammonia [109]. This combination achieves an energy requirement of 4.6 MJ·mol^−1^ NH_3_, which is over four times less than the state-of-the-art plasma-enabled ammonia synthesis from N_2_ and H_2_ with reasonable yields (>1%). However, this chemistry, although interesting, is not relevant for the scope of this review.

A possibility to improve the performance of NTP is to combine it with a catalyst (plasma catalysis). The problem is that the mean free path of a gas molecule is about three to four orders of magnitude shorter than the typical distance from where it is generated to the catalyst surface in usual plasma catalysis reactor configurations. Therefore, when N_2_* species are formed in the plasma, they collide with many other gas-phase molecules (10^2^–10^5^ depending on the pressure) and dissipate the energy acquired from the plasma before contacting the catalyst. The possibility to generate the plasma at the surface or even within the catalyst itself (micro-discharge) should thus be realized [21]. The second issue is that the catalyst should avoid quenching of the generated excited species and react synergistically to control the path of transformation. There are thus still major challenges to develop more effective systems for NH_3_ synthesis by plasma catalysis, but from the perspective of this contribution, the point is to understand which paths have been proposed for ammonia transformation on the surface of the catalyst interacting with N_2_* species and thus when the first step of N_2_ molecule activation (the usual limiting step at temperatures close to ambient) is overcome by the effect of plasma. Carreon [24] recently summarized the studies on the surface mechanisms proposed in plasma catalysis for N_2_ fixation. Although there are discordances, the results can be summarized as follows, depending on the pressure of the operation:

- in vacuum plasma, the atomic N species (generated by N_2_ dissociation in the plasma) are adsorbed on the catalyst surface and react with atomic H from the gas phase to form NH(s) species and finally ammonia; the rate limiting step is the surface reaction;
- in atmospheric pressure plasmas, the mechanism is still controversial. One possibility is the dissociative adsorption of excited N_2_ as the first reaction step but followed by surface dissociation and then surface hydrogenation by chemisorbed atomic hydrogen species. The other possibility is that atomic nitrogen directly generated in the plasma is chemisorbed and hydrogenated on the surface.

Thus, the mechanisms proposed are close to those in thermal ammonia synthesis catalysis because the main limit of activation of the N_2_ molecule is overcome by the plasma activation. Note that these mechanisms are valid using H_2_ directly, but become less clear when water is instead present as a hydrogen source.

## 7. Conclusions

The analysis of the molecular mechanisms of solar NH_3_ production shows that there are two groups of mechanisms. The first one occurs either at a high temperature (conventional thermal catalysis) and in plasma catalysis where the break of the *n* ≡ *n* bond is the first stage, followed by hydrogenation of the nitrogen atoms or NH_x_ species. The second group of mechanisms, based on the direct hydrogenation of undissociated N_2_ molecule, occur in electrocatalysis, enzymatic catalysis (nitrogenase) and for the most active metal complexes. We have not specifically analyzed photocatalysis for sake of conciseness here, but mechanistic indications are well in line with those discussed for electrocatalysis. These two groups of molecular mechanisms of N_2_ fixation have also been analyzed in various recent reviews, but the point made here is that the consequences of the different initial steps in N_2_ fixation are rarely then correctly accounted for in the proposed mechanisms of reaction.

Attention has been given here to remarking on the key features of the proposed mechanism of nitrogenase, which can be summarized as: (i) need for a multi-electron and -proton simultaneous transfer, not as sequential steps, (ii) the formation of bridging metal hydride species, (iii) the formation of intermediates stabilized by bridging multiple metal atoms and (iv) capability of the same sites to be effective both in N_2_ fixation and in CO_x_ reduction to C2+ products. A biomimetic electrocatalyst (or photocatalyst) should thus be able to have all these features. The only electrocatalyst we have identified, as far as we know, is based on iron oxide/hydroxide nanoparticles stabilized at defect sites of a nanocarbon support.

In principle, other types of mechanisms could also be effective. However, we have also evidenced here, even if not through a systematic analysis, that there is a very large discordance in the proposed mechanisms for both N_2_ fixation and CO_2_ multi-carbon (C2+) reduction, despite the limited differences in the performances which contradict the large differences in the reaction mechanisms and nature of the active sites. Therefore, at least in terms of the current stage of development, it is rather difficult to identify a mechanism which could be the basis for the further development of these (electro)catalysts, while a breakthrough change is required to go from the current to the needed performances for the industrialization of the process.

We feel that the biomimetic approach outlined above, and specifically the capability to realize multi-electron/proton simultaneous transfers and activity both in N_2_ fixation and CO_2_ to C2+ reduction, could be a relevant source of inspiration to develop next-generation electrocatalysts, which are needed to address the challenging transition to a future of sustainable energy and chemistry beyond fossil fuels.

## Data Availability

Data sharing not applicable.

## Abbreviations

ATP	Adenosine triphosphate
CNT	Carbon nanotube
CO_2_RR	CO_2_ reduction reaction
DFT	Density functional theory
GHG	Greenhouse gas
HB	Haber–Bosch
NHE	Normal hydrogen electrode
NTP	Non-thermal plasma
NRR	Nitrogen reduction reaction
PCET	Proton-coupled electron transfer
PEC	Photoelectrocatalytic
RE	Renewable energy
TON	Turnover number
